# Clinical Use of Blood Flow Analysis through 4D-Flow Imaging in Aortic Valve Disease

**DOI:** 10.3390/jcdd10060251

**Published:** 2023-06-09

**Authors:** Omer Mansoor, Julio Garcia

**Affiliations:** 1 Undergraduate Medical Education, Cumming School of Medicine, University of Calgary, Calgary, AB T2N 1N4, Canada; mohammad.mansoor@ucalgary.ca; 2 Department of Radiology, Cumming School of Medicine, University of Calgary, Calgary, AB T2N 1N4, Canada; 3 Department of Cardiac Sciences, Cumming School of Medicine, University of Calgary, Calgary, AB T2N 1N4, Canada; 4 Stephenson Cardiac Imaging Centre, Libin Cardiovascular Institute, University of Calgary, Calgary, AB T2N 1N4, Canada; 5 Alberta Children’s Hospital Research Institute, Cumming School of Medicine, University of Calgary, Calgary, AB T2N 1N4, Canada

**Keywords:** cardiac flow, abnormal blood flow, 4D-flow MRI, valvular disease

## Abstract

Bicuspid aortic valve (BAV), which affects 1% of the general population, results from the abnormal fusion of the cusps of the aortic valve. BAV can lead to the dilatation of the aorta, aortic coarctation, development of aortic stenosis (AS), and aortic regurgitation. Surgical intervention is usually recommended for patients with BAV and bicuspid aortopathy. This review aims to examine 4D-flow imaging as a tool in cardiac magnetic resonance imaging for assessing abnormal blood flow and its clinical application in BAV and AS. We present a historical clinical approach summarizing evidence of abnormal blood flow in aortic valve disease. We highlight how abnormal flow patterns can contribute to the development of aortic dilatation and novel flow-based biomarkers that can be used for a better understanding of the disease progression.

## 1. Introduction

In the cardiovascular context, abnormal blood flow through a vessel is often due to a disease process [1]. Abnormal and/or aberrant blood flow is often contrasted with laminar flow, which refers to a streamlined movement of blood through vessels seen in relatively healthy patients [2]. The random nature of abnormal blood flow can disturb the underlying endothelium within the vessel and lead to vascular remodeling [3]. These additional stresses to a blood vessel from an abnormal flow can potentially increase aortic complications resulting from certain valvular diseases. Understanding abnormal blood flow, its mechanism, and how it is quantified can provide key insight into how valvular diseases are understood.

The development of abnormal blood flow has been demonstrated to be due to a variety of pathological causes, such as atherosclerotic plaque or valvular disease [4]. Altered blood flow increases physiologically with age in the ascending aorta due to aortic dilation and irreversible pressure loss, which facilitate the occurrence of abnormal flow [5,6,7]. Quantifying this measure can help distinguish age-related changes from pathological diseases and assist clinical outcomes by providing additional information related to outcomes, tracking management, and overall disease progression [7].

**Figure 1:**
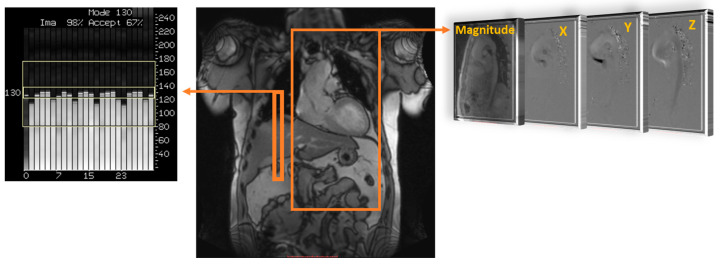
Data acquisition of 4D flow. Coverage of the region of interest; in this example, the thoracic aorta (large orange rectangle) is acquired using electrocardiographic gating and respiratory control to reduce motion artifacts, using diaphragm navigator gating (small orange rectangle). Velocity encoding (right side) obtains blood flow velocities along all three spatial dimensions (X, Y, and Z) and anatomy magnitude over the cardiac cycle.

Understanding how blood flow is measured through current non-invasive technology is important for its use in clinical environments. Traditionally, cardiovascular magnetic resonance imaging (MRI) has provided high-quality imaging of the heart with its surrounding structures. Recent advancements in MRI sequences and technology facilitate the quantification of blood flow [8,9,10]. Historically, phase contrast MRI has extended quality images to include angiography and outline flow within blood vessels. Velocity has only been measured in-plane, perpendicular to a blood vessel, such as through a Doppler ultrasound. By using a phase-contrast approach and applying three-dimensional velocity-encoding, Figure 1, we can capture both blood flow velocity and the heart’s anatomy [9]. This MRI technique is called ‘time resolved’ three-dimensional MRI, commonly known as 4D-flow MRI, which also allows additional flow characteristics to be quantified [11]. Moreover, advanced 4D-flow-based parameters can be extracted, for example, turbulent kinetic energy (TKE), which has been compared in research studies and used in risk stratification, disease outcomes, and potential changes in management [5,6]. TKE describes the kinetic energy of the fluctuating velocity field, which is computed using the intravoxel velocity standard deviation from the velocity magnitude images [12].

**Figure 2:**
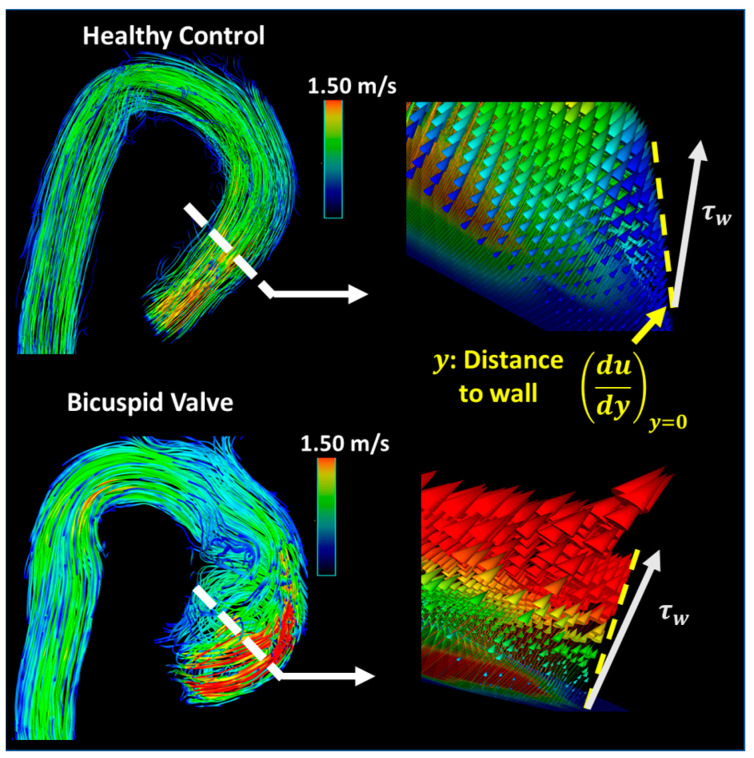
Wall shear stress calculation. The white dashed lines represent the location where the sample velocity profile was obtained. From these flow profiles, the shear stress rate and blood flow spatial deformation, illustrated by the gold lines, can be estimated. The near wall region is the boundary layer where the wall shear stress (WSS) forces occur. The WSS expresses the force per unit area exerted in the fluid direction on the local vessel tangent ($\tau_{w}$). (**Top**): a healthy control illustrated the normal flow. (**Bottom**): bicuspid aortic valve illustrated the altered WSS produced via a right–left cusp fusion.

Wall shear stress (WSS) is closely related to blood flow development at the vessel wall level, whether the flow is normal or abnormal, as shown in Figure 2. WSS measures the amount of force exerted on the wall of the vessel [13]. WSS_peak_ indicates the highest WSS experienced in the vessel, typically assessed at peak systole. For example, aortic WSS was demonstrated to be elevated in different valvular pathologies, such as a bicuspid aortic valve (BAV) and aortic stenosis (AS) [14,15,16,17].

In addition to WSS, another value that could be useful is flow displacement, which measures the direction of the blood flow jet coming into the aorta and examines how off from mid-line it is. Flow displacement was defined as the distance between the center of the lumen and the “center of the velocity” of the forward flow and was normalized to the lumen diameter [18]. Valvular flow displacement can also characterize the displacement of the vena contracta, where the highest transvalvular blood flow velocity can be observed [19,20]. This directional change in blood flow can provide a quantifiable outcome measure after repair to ensure the direction of flow is repaired. Apart from WSS and flow displacement, TKE obtained from 4D-flow MRI measures how much energy is lost to turbulent blood flow in the form of heat [12,14]. As blood flows in a complex turbulent and random nature, it dissipates its energy to the vessel more so than in the laminar fashion. As a result, the TKE measure aims to quantify turbulence from an energy perspective and indicate work loss because of non-laminar flow transition through the blood vessels.

BAV is a common congenital heart disease with a prevalence of 1% of the population [21]. In this condition, only two leaflets are present on the aortic valve as opposed to three. As a result, abnormal blood flow occurs across the valve leaflets and downstream the aorta. Furthermore, about 50% of BAV patients develop aortic coarctation, which consists of a discrete stenosis or hypoplastic segment located most often immediately after the left subclavian artery [22]. Although aortic valve stenosis and regurgitation are the most common complications of BAV, the dilation of any or all segments of the aorta (also known as aortopathy) is present in 50% of BAV patients [23]. Along with abnormal blood flow, one of the main reported associations between BAV, aortic coarctation, and aortic dilation is the impairment of the endocardial–mesenchymal transition in the homozygous NOTCH1 mutation [24,25,26]. Quantifying abnormal flow could help with management and/or prognosis. Different imaging techniques aim to capture blood flow and quantify it in BAV. Currently, transthoracic echocardiography (TTE) remains the gold standard for diagnosing BAV pathology. If the leaflets cannot be visualized on TTE, additional imaging, such as cardiovascular magnetic resonance imaging, can be used. Other techniques, such as gadolinium enhancement in CMR imaging, can be used to assess fibrosis in the myocardium of the heart and for aortic stenosis patients.

This review aims to examine 4D-flow imaging as an advancement in cardiac MRI, how abnormal blood flow can be quantified, and its clinical application in two different valvular pathologies: bicuspid aortic valve (BAV) and aortic stenosis (AS). Additionally, this review will provide potential future directions after appraising the current research in this area.

## 2. Cardiovascular 4D-Flow

**Figure 3:**
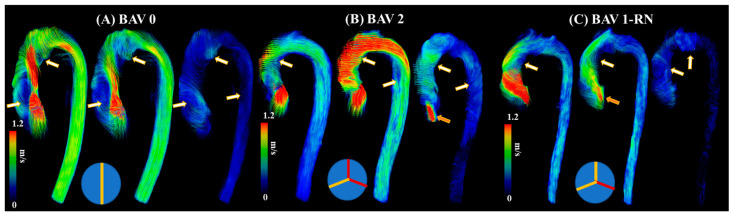
Examples of abnormal blood flow in bicuspid valve disease. Panel (**A**) illustrates a true bicuspid valve (schematic at the bottom), panel (**B**) illustrates a type 2 valve fusion phenotype (schematic at the bottom, red lines correspond to valve fusion), and panel (**C**) illustrates a type 1 valve fusion phenotype (schematic at the bottom). White-gold arrows point to regions with abnormal blood flow patterns at systolic acceleration, peak systole, and systolic deceleration. Orange arrows point to valvular regurgitation or insufficiency.

The current literature describes a novel understanding of measuring abnormal blood flow using 4D-flow imaging and key flow parameters closely associated with it. The process of obtaining hemodynamic measurements with 4D-flow imaging has been well defined in the last decade. Standard-of-care 2D phase contrast MRI (PC-MRI) measures thought plane velocity and fails to capture the complex nature of abnormal blood flow. It has the advantage of having a short acquisition of ~20 s. For 4D-flow imaging, however, the acquisition is longer, 10–20 min, using basic acceleration protocols [8,11,13]. The reason behind the additional scan time is the volumetric velocity acquisition of the blood flow in three dimensions, as opposed to one direction seen in 2D PC-MRI [14]. As a result, 4D flow captures the complex nature of the cardiovascular blood flow, as shown in Figure 3.

## 3. Clinical Applications in Bicuspid Valve Disease

The initial clinical observations were mostly focused on qualitative imaging characteristics produced by BAV. The qualitative assessment included classification schemes for aortic dilation and valve–fusion morphology. A key milestone in understanding the relationship between abnormal blood flow and different aortic pathologies is the advancement of imaging techniques to assess blood flow velocities. The flow across the aorta is currently examined with single-direction velocity; however, 4D-flow imaging allows 3D velocity measurements to quantify blood flow in various directions. For hemodynamic imaging, characteristics signal loss patterns and abnormal blood flow patterns (e.g., helicity and vorticity) were mostly reported. Their main limitations remained the subjectivity of the evaluation and high observer variability. The development of BAV aortopathy has been attributed to genetic and hemodynamic bases. The latter motivated the introduction of quantitative hemodynamic approaches. Two of the most recurrent markers are WSS and flow displacement. WSS has been extensively evaluated and was expected to bring an association with the extracellular matrix in the aorta, resulting in matrix disruption and elastin fragmentation with increased WSS. The main limitation of WSS, as derived from 4D flow, is the limited spatial resolution near the wall (~2.5 mm by voxel). Flow displacement was introduced as a simplified metric of abnormal flow patterns while ensuring the capture of the maximum blood flow displacement and providing a simple way to replace the qualitative assessment of blood flow patterns.

One of the first studies outlining abnormal blood flow in BAV using MRI was performed by Rees et al. [27]. In this study, thirty-six patients were examined after aortic coarctation repair using different imaging modalities. Although abnormal blood flow was not the main objective of this study, it does illustrate the early usage of MRI technology to examine signal loss as a potential marker for blood flow. Six patients from seven demonstrated marked systolic signal loss, which was attributed to the high velocity at the isthmus after the repair of the coarctation. Four additional patients demonstrated abnormal blood flow across the aortic valve, which was presumed to be due to a BAV. This study did introduce the link between abnormal blood flow and signal loss on MRI.

One primary question was to demonstrate why a two-leaflet valve has a higher fail rate than a three-leaflet valve [28]. This study highlights abnormal blood flow as an underestimated factor in BAV disease progression because it can induce irregular patterns of opening and closing of the valve. This irregular pattern can predispose the valve to fibrosis and calcification. This work also illustrates how abnormal blood flow passed the valve can influence the aortic wall and, over time, lead to dissection and dilation. The authors mostly examined preserved BAV from deceased patients that had molds created and were examined in a simulator. The only imaging conducted was ultrasound. Robicsek et al. [28] provide a pathophysiological basis for abnormal blood flow across BAV and the subsequent clinical implications of having two leaflets.

**Figure 4:**
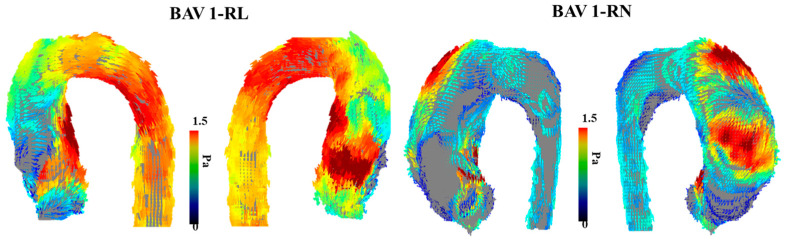
Examples of wall shear stress in bicuspid aortic valve type 1 phenotype. The (**left**) panel illustrates the vectorial wall shear stress (WSS) representation of a bicuspid aortic valve (BAV) patient with type 1 right–left leaflet fusion. The (**right**) panel illustrates the vectorial WSS representation in a patient with type 1 right non-coronary fusion. The affected region within the aorta is closely related to the eccentric systolic blood flow jet produced by each valve phenotype.

An increasing amount of evidence showcases that BAV disease and its associated aortic complications, such as dilation, occur even in hemodynamic normal BAV [29]. Abnormal or aberrant blood flow might not be the cause of the downstream aortic effects of having a BAV. However, a case report performed by Hope et al. outlines the first direct approach to using 4D-flow imaging to specifically examine abnormal blood flow and flow parameters in a 14-year-old child with BAV [30]. The patient had aortic coarctation, and the authors hypothesized that increased hemodynamic load is placed on the proximal aorta due to the BAV, which leads to the eventual dilation of the aortic wall. The article highlights abnormal blood flow as a future area of research along with WSS using 4D-flow imaging. Furthermore, another study from the same group explores the clinical application of 4D-flow imaging in BAV [31]. This study builds upon the intrinsic wall or abnormal blood flow pattern theory of the aortic dilation eventually seen in BAV patients. This article argues that different disease progress happens in BAV patients based on how the two leaflets of the aortic valve are fused or what BAV phenotype the patient has. As a result, it cannot be explained by just intrinsic features of the aortic wall becoming dilated, and additional flow parameters should be at play. This work points to increased hemodynamic stress being linked to smooth muscle cell apoptosis. This article does provide more power than previous studies by being a randomized control trial and having a higher sample size, which provides more validity to the reported results and findings that agree with previous studies [32]. In addition, BAV patients with eccentric systolic blood flow patterns that are off center and towards the wall of the aorta were statistically significantly increased in terms of their WSS values compared to BAV patients with normal or mild eccentric flow patterns. Figure 4 illustrates the impact of eccentric systolic blood flow on WSS in RL and RN BAV types.

The reported findings support a flow-mediated process for patients with BAV aneurysms; however, it does outline the evidence for abnormal wall properties. This article supports a dual theory where the wall of the aorta and flow dynamics both play an important role in dilating the aorta in patients with BAV to predispose them to an aortic aneurysm.

Previous research suggests that WSS derived from 4D-flow correlates with aortic elastic fiber thinning in BAV [33]. BAV patients have abnormal WSS values proximal in the aorta compared to patients with normal, tricuspid aortic valves. The increased WSS, when measured non-invasively via 4D-flow MRI, provides more evidence that flow hemodynamics play a role in aortic dilation in these patients as opposed to just an intrinsic feature of the aortic wall. One area of concern in this study was that 96% of the BAV patients also had aortic stenosis and/or aortic regurgitation, which could be a concurrent pathology for the WSS hemodynamic-mediated aortic thinning. The correlation of WSS measures with aortic pathology provides a different insight into abnormal blood flow’s role on BAV and further validates WSS as a clinical marker in relation to aortic thickness. The article highlights that the results might not be sensitive enough to discern between a moderate and a significantly dilated aorta.

Meierhofer et al. [34] provide a strong prospective study design that outlines how significant flow parameters differ in patients with BAV and those with tricuspid valves. One of the strengths of the article is the strict inclusion criteria—this provides a strong foundation for reducing confounders and ensuring accurate patient population representation. The reduction of confounders did not seem to be as apparent of a priority in previous studies on 4D-flow imaging in BAV patients. The article argues that WSS and flow patterns can risk stratifying patients with BAV to have an aneurysm as opposed to an aortic diameter. This is in line with the risk stratification performed by Hope et al. [31]. With elevated WSS in healthy BAV patients with normal aortic diameters, this article does provide an additional way to identify the patients most likely to develop an aneurysm.

**Figure 5:**
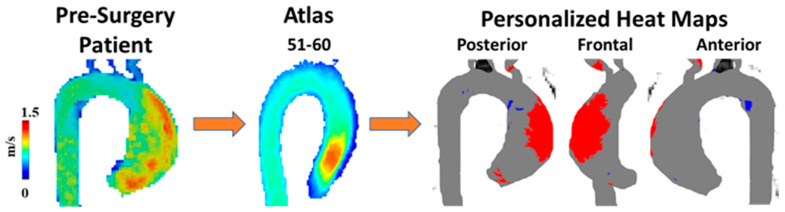
Example of wall shear stress heat map. A 60-year-old man with a bicuspid aortic valve (BAV) was scanned prior to surgery using 4D-flow MRI. Patient’s velocity field was matched by age and biological sex to a velocity atlas allowing to identify abnormal regions of wall shear stress using heat maps. Red in heat maps represents high wall shear stress, blue represents low wall shear stress.

A unique approach to utilizing WSS for patients with BAV was presented by van Ooij et al. [35]. As opposed to only quantifying WSS, the goal of this article was to develop individualized ‘heat maps’ that would map abnormal WSS values in an easy-to-read format. The control group provided the normal values of the WSS and if areas of the aorta in a patient with BAV fell outside (mean + 1.96·SD), they were highlighted in red, Figure 5. These maps were also correlated with aortic geometry to account for variations. Most aorta showcased abnormal WSS values in the greater curvature of the ascending aorta. One important finding in this article was that there was no significant correlation between the abnormal WSS surface percentages and other cardiac markers (cardiac output, heart rate, or body surface area). This indicates that abnormal WSS may be an independent marker from standard-of-care imaging cardiac metrics. These correlations could be outside the scope of certain articles or not provide additional value in the setting of BAV patients; however, understanding how 4D-flow measures compared to basic cardiac findings could prove useful.

Although much of the previous research has focused on adults and age-matched controls for BAV disease, Rose et al. [36] examine this area in pediatric and young adult populations. Although BAV can be asymptomatic within patients’ childhood and adolescent life stage, they are at risk of complications discussed before, such as aortic dilation or aortic aneurysm. The main goal of this article is to examine these hemodynamic values in BAV patients using 4D-flow imaging and understand if they change over time in a pediatric and younger population. The results showcase that no significant change in WSS and peak velocity occurred over a mean follow-up time of 1.8 ± 1.0 years. There were no significant correlations between WSS and aortic diameter, a contrast to previous studies in this review but in line with Hope et al. [37]. Although there have been discrepancies in the literature regarding WSS and aortic dilation in adult patients, most articles indicate a correlation. Rose et al. [36], however, do not describe this correlation in pediatric and younger patients. This could be attributed to the small follow-up time and how WSS and its effect on the aortic wall could need much more time to occur. A key finding in younger patients with BAV is the stable hemodynamic parameters (WSS and peak velocity) despite increased ascending aortic growth (i.e., diameter increment over time, typically assessed on a one-year basis). These findings, however, need to be taken into the context of the limitations of this study. The small follow-up time is a significant limitation of this study as hemodynamic changes could take a much longer time to create any clinical effect. Although this study has a smaller sample size, the power calculations indicate they should be able to detect clinically significant results in velocity and WSS.

**Figure 6:**
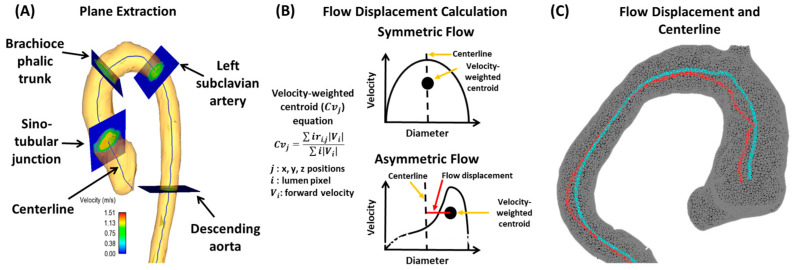
Example of flow displacement calculation. Panel (**A**) shows the plane analysis extraction from a centerline. Multiple equidistant planes are automatically generated for analysis. Landmark planes help to characterize the aorta in a reproducible manner. Panel (**B**) illustrates the calculation performed on each plane to obtain flow displacement. Panel (**C**) shows an example of flow displacement (red line) and centerline (blue line) in a bicuspid valve patient with aortic dilation.

Burris and Hope [32] agreed with Borger and David’s [29] analysis by reflecting on whether abnormal blood flow or the aortic wall characteristics causes the aortic consequences of BAV, and they agreed that it is debated in the literature. This work mainly discussed WSS and flow displacement, as shown in Figure 6. WSS estimates the frictional force applied onto the endothelium from flow viscosity, with higher WSS values indicating remodeling of the endothelium vasculature. Burris and Hope [32] outline how flow displacement can also distinguish between the types of BAV and go further to contrast previous studies to mention how flow displacement can be measured using current 2D PC-MRI. This provides a different perspective from previous articles that have not mentioned obtaining this parameter with 2D PC-MR imaging. Aside from flow displacement, a key theme throughout this article is that these parameters could become clinically relevant in the future. As 4D cardiac MRI is researched further and larger datasets are collected, we will understand the clinical relevance of this imaging technique.

The reproducibility of 4D-flow imaging in BAV patients is key to understanding its relationship to the disease. Hope et al. demonstrated that peak velocity, flow displacement, and WSS were consistently reproducible [37]. Flow displacement was found to correlate with aortic growth, which refers to the dilation of the ascending aorta in patients with BAV. WSS, however, did not correlate well with aortic growth, indicating a mismatch between this value and its contribution to aortic growth, as outlined in other articles. The authors reconcile this by attributing it to the technological limitations of MRI, as opposed to a true correlation deficit between WSS and aortic growth. This article provides future directions in outlining how flow displacement should be used as a potential clinical marker for risk-stratifying patients with BAV.

Lewandowski et al. [38] outline a more practical approach to translating 4D-flow imaging into clinical practice and usability with clinicians. The article agrees with previous sentiments around the impact of quantifying abnormal blood flow for understanding cardiovascular pathology. Accurate measurements of abnormal flow often require an invasive approach; however, 4D-flow imaging could provide a safer alternative to obtain these values. This article highlights how 4D-flow imaging needs to be standardized in how the parameters are acquired, which would reduce variability in how fast these measurements are obtained. This distinction is important from Burris and Hope [32], who outlined how fast 4D-flow imaging has become, but not necessarily how widely available or adopted it is in imaging centers worldwide. By automating analysis and having standardized reporting protocols, 4D-flow imaging could obtain a systematic approach that would be useful in clinical trials and analyzing large amounts of data. This would provide a basis to understand if abnormal blood flow could predict disease outcomes, specifically in BAV.

Apart from younger patients, 4D-flow imaging can play a role in follow-up for BAV repairs, as outlined by Lenz et al. [39]. With certain advantages of aortic valve repair over prosthetic valve replacement, clinical markers of hemodynamics after surgery can be quite valuable. Flow displacement was examined and became centrally located within the vessel after aortic valve repair in 60% of patients. Other hemodynamic parameters, such as global and regional WSS, became significantly reduced after surgery in BAV patients. In comparing this study to previous articles and research in the area, mortality correlation could be a useful clinical indicator to understand in relation to flow parameters. More recently, Soulat et al. [40] examined aortic coarctation repair in BAV patients using 4D-flow imaging for a median of four years after repair. This study found that 4D-flow parameters were stable between serial scans. Not only does this provide a non-invasive way of examining blood flow, but it reaffirms the surgical approach to BAV patients’ repair of aortic coarctation. When valve replacements occur or aortic complications are repaired, 4D-flow imaging can provide a non-invasive approach to blood flow hemodynamics and characterize local changes. Beyond surgical repairs for BAV patients, more studies need to examine mortality correlation from BAV patients’ blood flow values measured via 4D-flow imaging. Although flow displacement has been demonstrated as a better alternative for risk-stratifying patients with BAV and the complication of aortic dilation by Burris and Hope [32], understanding if measuring at more regular intervals for higher-risk patients provides a mortality benefit would better outline the clinical value of 4D-flow imaging. This could provide direction for future research studies in 4D-flow imaging for BAV patients.

## 4. Clinical Applications in Aortic Stenosis (AS)

Aortic stenosis (AS) refers to the narrowing of the aortic valve, often due to calcification or other causes, which restricts blood from entering the aorta [5]. Patients can be stratified into mild, moderate, or severe based on their symptoms, ejection fractions, and transthoracic echocardiography (TTE) findings. This section aims to analyze articles that have examined AS and 4D-flow imaging.

**Figure 7:**
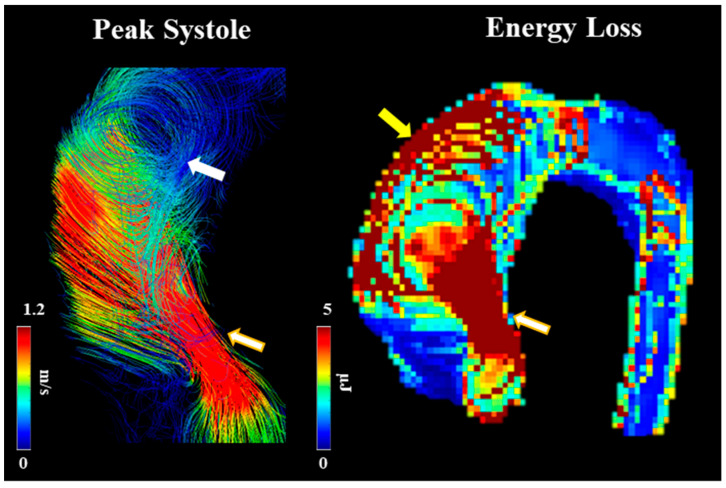
Example of energy loss in a subject with bicuspid valve and moderate aortic stenosis. White arrow points to helical flow. Yellow arrow points to energy loss at the aortic wall. Golden-white arrows point to transvalvular energy loss.

Barker et al. [41] outline an initial approach to examining abnormal blood flow and energy loss using 4D-flow imaging. They examined laminar flow energy loss from different aortic pathologies, including AS to provide in vivo results. The researchers had two patient groups (dilated ascending aortas and those with AS with dilation), which were picked because of their association with abnormal blood flow. The article outlines how viscous energy loss measured by 4D-flow imaging could become a useful parameter in the future as it produces one value if one segment needs to be examined (E_L_′). This could be useful to clinicians as one value provides the opportunity to discuss between patients, risk stratification, and use in clinical research projects later on. Since this article positions itself as a pilot study, it does provide a basis for future research studies to be conducted on how 4D-flow imaging can be useful in defining AS patients, their disease management, and their disease outcomes. An example of energy loss is shown in Figure 7.

Few studies have simultaneously assessed AS severity using 4D-flow imaging and transthoracic echocardiography (TTE). Binter et al. [5] examine AS severity using TTE and 4D-flow imaging to examine if additional turbulence parameters provide clinical benefit. One key finding was that TKE was found to be significantly higher in AS patients but did not correlate to any TTE parameters. The results indicate that TKE could provide supplementary information to TTE to help stratify patients based on the severity of AS. TKE could be used as a measure of exercise-induced symptoms but is not an option for certain patients to help place their AS into mild, moderate, or severe categories. The article provides appropriate future directions for the research of 4D-flow imaging and AS by highlighting how TKE could be correlated with certain laboratory values that predict outcomes in patients. Understanding the clinical use of TKE as a potential predictor of mortality could provide the basis for 4D-flow imaging to become more involved in the diagnosis and management of AS, similar to BAV. If TKE could be used to stratify AS patients without relying on symptoms, which are often subjective, 4D-flow imaging could provide useful clinical information. One area the article could have focused on more was the outcome of AS, such as dilation or LV hypertrophy, and how TKE values change with respect to these downstream complications. This approach would have required longer follow-up time and ensuring patients enrolled did not already have these complications and/or are minimized during inclusion criteria.

Adriaans et al. [42] provide a prospective study to further outline the role of 4D-flow imaging in evaluating AS. One key finding is that although 2D PC-MRI is more readily available, it does underestimate fluid hemodynamic values compared to TTE in the context of AS. However, 4D-flow imaging more accurately reflected and could overestimate the values obtained from TTE, such as peak jet velocity. The article also highlights the mechanism for why TTE can underestimate peak velocity. Due to eccentric flow and resultant flow displacement, the blood passing through the aortic valve can be displaced due to stenosis. Since the ultrasound beam for TTE is user dependent, and the velocity is off center, this misalignment between the TTE beam and the flow direction of blood can underestimate certain values. As the stenosis gets more severe, the underestimation using TTE worsens. This underestimation using TTE, however, most likely does not affect clinical outcomes because TTE is still used as the mainstay for AS diagnosis, and any deviation is only being measured given that 4D-flow imaging can measure velocity in multiple directions and is not user dependent. As a result, any deviation due to the flow displacement is incorporated into the TTE, which is the standard used to grade the severity of AS. The study also touches on how processing times and advanced MR imaging technology prevents the widespread adoption of 4D-flow imaging, even though it does have the potential to provide useful clinical information. The results highlights that 4D-flow imaging is far from replacing TTE as the standard due to adaptation limitations and other constraints but, rather, only provides supplemental information.

## 5. Conclusions and Future Directions

Ultimately, 4D-flow imaging is an advanced cardiac MR imaging technique that allows fluid hemodynamics to be measured non-invasively in patients. As a growing field in radiology, its adaptation and clinical use remain underutilized. Apart from flow displacement, WSS has been a consistent measure in BAV patients using 4D-flow imaging. Different studies have demonstrated how, over time, peak systolic WSS mean magnitude on the aortic wall can lead to endothelial dysfunction and eventual thinning of the wall [43]. There does exist a debate in the literature on whether the thinning of the aorta is due to an intrinsic property of the wall itself (such as genetics, underlying hereditary disease, and connective tissue disorders) or repeated stress on the wall from abnormal blood flow onto the aorta from a bicuspid valve. Most articles analyzed here agree with a combined theory that along with intrinsic factors of the aortic wall, WSS contributed significantly to aortic dilation in patients with BAV as measured with 4D-flow MRI. Surgical repairs of BAV also proved to provide a significant reduction in these measures. Although 4D-flow MRI can showcase an exciting new area of imaging and more quantifiable, objective measures of blood flow, more studies are needed to understand how to change BAV management beyond risk stratification and examine mortality. Some studies examined here had short follow-up times, which may have not provided the necessary time to see a significant impact or understand the full scope of these fluid dynamics on wall endothelial function. With improved technology and additional research studies, 4D-flow imaging could become a key determinant in BAV patients’ management and follow up.

For AS, 4D-flow imaging could play an important role in disease severity. For example, Binter et al. [5] demonstrated how 4D-flow imaging can be used to supplement the findings from TTE for AS to distinguish between moderate and severe disease. The outcomes from the literature for AS reflect similar findings for BAV disease, where both have demonstrated that 4D-flow imaging can play a role in defining and risk-stratifying patients. Another area for future directions is to examine TKE measurements using 4D-flow imaging before and after interventions to treat AS. This could provide a non-invasive approach to understanding how abnormal blood flow changes with intervention, specifically within the context of AS. With how common AS has become in recent years and in developed countries, understanding the change in turbulence blood parameters with intervention could provide useful clinical information on successful treatment. A summary of the main articles presented in this review is presented in Table 1.

In conclusion, 4D-flow imaging is headed in the right direction and provides useful, but often, supplemental information to BAV and AS diseases. With even more technological advancements, quicker acquisition times, and more widespread adaptation, 4D-flow imaging could become a mainstay in the diagnosis, management, and risk stratification of valvular pathologies.

## Figures and Tables

**Table 1:** Article summary.

Studied Population	Key Parameters	Reported Findings
Soulat et al., 2022 [40] *n* = 15 BAV Age = 35 ± 8 years	PV, WSS, PWV	WSS is stable over time in BAV patients. Increased PV at coarctation.
Adriaans et al., 2020 [42] *n* = 20 Age = 69.3 ± 5 years	PV, pressure, AVA	AS revealed higher flow velocities than TTE.
Lenz et al., 2020 [39] *n* = 20 Age = 35 (IQR 29–47)	Flow displacement, WSS	Flow displacement and WSS were reduced after valve repair.
Rose et al., 2019 [36] *n* = 19 Age = 14.0 ± 5.7 years	PV, WSS, aortic Z-scores	Stable hemodynamic markers over short-term follow-up despite significant aortic growth using Z-scores. Baseline aortic PV was a predictor of aortic dilation.
Bollache et al., 2018 [33] Patients: *n* = 27 Age = 52 ± 15 years Controls: *n* = 20 Age = 48 ± 14 years	WSS, Elastic fibers size, Aortic diameter	BAV showed increased WSS associated with elastic fiber thinning. Elastic fiber thinning correlates with impaired tissue biomechanics.
Binter et al., 2017 [5] Patients: *n* = 51 Age = 67 ± 15 years Controls: *n* = 10 Age = 69 ± 5 years	TKE	Elevated TKE implies higher energy losses associated with BAV. TKE may help to distinguish within the heterogeneous population of patients with moderate to severe AS.
van Ooij et al., 2015 [35] Patients: *n* = 13 Age = 51 ± 17 years Controls: *n* = 10 Age = 50 ± 14 years	WSS	Elevated WSS was elevated in the ascending aorta and correlated with PV.
Barker et al., 2014 [41] Patients with aortic dilation: *n* = 16 Age = 52 ± 8 years Patients with AV stenosis (age-sex matched): *n* = 14 Age = 46 ± 15 years Controls: *n* = 12 Age = 37 ± 10 years	Viscous energy loss	Viscous energy loss was significantly elevated in the thoracic aorta for patients with dilated aorta and patients with aortic stenosis compared to healthy volunteers. Viscous energy loss in patient cohorts was significantly elevated and indicates that cardiac afterload is increased due to abnormal flow.
Hope et al., 2014 [37] Patients: *n* = 13 Age = 26.5 (17–43) years Patients: *n* = 12 Age = 30.7 (17–64) years	Flow displacement, WSS	Flow displacement is a simple and reproducible hemodynamic marker that shows good correlation with aortic growth in patients with BAV.
Meierhofer et al., 2013 [34] Patients: *n* = 18 Age = 25 (10–44) years Controls: *n* = 18 Age = 25 (8–42) years	WSS	WSS and flow patterns in the ascending aorta in BAV patients without concomitant valve or vessel disease are significantly different compared with tricuspid aortic valve. Higher shear forces may have an impact on the development of aortic dilation in patients with BAVs.
Hope et al., 2011 [31] BAV patients with normal flow: *n* = 7 Age = 20.4 ± 7.9 years BAV patients with abnormal flow: *n* = 19 Age = 30.5 ± 12.6 years Controls: *n* = 20 Age = 26.9 ± 10.4 years	WSS, Eccentric Flow	BAV and eccentric systolic blood flow was found to have elevated WSS.

PV: peak velocity; WSS: wall shear stress; PWV: pulse wave velocity; AVA: aortic valve area; TKE: turbulent kinetic energy; BAV: bicuspid aortic valve.

## Data Availability

The anonymized data presented in this study are available upon request from the corresponding author. The data are not publicly available due to privacy and ethical restrictions.
